# Exploration and analytical techniques for membrane curvature-sensing proteins in bacteria

**DOI:** 10.1128/jb.00482-24

**Published:** 2025-03-26

**Authors:** Takumi Komikawa, Mina Okochi, Masayoshi Tanaka

**Affiliations:** 1 School of Materials and Chemical Technology, Institute of Science Tokyo 163706, Yokohama, Kanagawa, Japan; 2 School of Materials and Chemical Technology, Institute of Science Tokyo 538511 , Meguro, Tokyo, Japan; Queen Mary University of London, London, United Kingdom

**Keywords:** biological membranes, membrane curvature-sensing proteins, *in vitro* assay, live cell assay, exploration assay

## Abstract

The mechanism by which cells regulate protein localization is an important topic in the field of bacterial biology. In certain instances, the morphology of the biological membrane has been demonstrated to function as a spatial cue for the subcellular localization of proteins. These proteins are capable of sensing membrane curvature and are involved in a number of physiological functions such as cytokinesis and the formation of membrane-bound organelles. This review presents recent advances in the *in vitro* evaluation of curvature-sensing properties using artificially controlled membranes and purified proteins, as well as microscopic live cell assays. However, these evaluation methodologies often require sophisticated experiments, and the number of identified curvature sensors remains limited. Thus, we present a comprehensive exploration of recently reported curvature-sensing proteins. Subsequently, we summarize the known curvature-sensing proteins in bacteria, in conjunction with the analytical methodologies employed in this field. Finally, future prospects and further requirements in the study of curvature-sensing proteins are discussed.

## INTRODUCTION

Biological membranes are primarily composed of phospholipids and proteins and serve a boundary to separate the cytoplasm from the external environment or define the compartment of membrane-bound intracellular organelles. Membrane morphology is precisely controlled and remodeled in response to physiological functions, and biological membranes exhibit distinct curved structures owing to the asymmetry of lipid composition or membrane proteins (1–3). In eukaryotes, several integral and peripheral membrane proteins are involved in membrane remodeling, including curvature-sensing proteins that preferentially interact with membranes having a specific shape or curvature. For instance, proteins containing the Bin/Amphiphysin/Rvs (BAR) domain are well known for their ability to sense and induce membrane curvature (4–6).

In contrast to eukaryotes, prokaryotes have historically been regarded as relatively simple structures, consisting of a plasma membrane that encapsulates soluble components such as proteins, DNA, ribosomes, and metabolites. However, in certain instances, prokaryotes also possess curved membrane-bound organelles (7). One illustrative example is the endospore of *Bacillus subtilis*, which is formed through forespore formation by asymmetric division and subsequent engulfment by the mother cell. During this process, a small amphipathic helical protein, SpoVM, is produced in the mother cell chamber and localized to the outer surface of the forespore; SpoVM then recruits other proteins to create the spore coat (8). SpoVM is known to recognize and localize to the positive membrane curvature of the forespore; it then recruits SpoIVA protein, which polymerizes to form a stable platform for the subsequent recruitment of coat proteins (9). Another organelle-like structure in prokaryotes is magnetosomes in *Magnetospirillum*, the membrane of which encapsulates 15–20 magnetite crystals (10). MamY, a key protein involved in magnetosome formation, exhibits weak homology with BAR domain-containing proteins and plays a role in cytoplasmic membrane constriction (11).

In addition to the formation of membrane-bound organelles, the localization of proteins to specific sites in the plasma membrane of prokaryotic cells contributes to dynamic subcellular transformations (12–14). For instance, the self-assembled cytokinetic protein FtsZ (Z-ring) spatially regulates cell division sites and recruits several proteins required for cytokinesis (15). FtsZ is directed to the future division site by the Min system, which is an important inhibitory system (16). In *B. subtilis,* the doublet DivIVA ring recruits MinC and spatially regulates the position of the FtsZ ring to the middle of the cell (17). DivIVA is a curvature-sensing protein, whose oligomeric clusters interact with and recognize negatively curved membranes (18, 19). Recently, purified FtsZ has been reported to exhibit curvature-sensing property (20). Understanding the structure and function of these cytoskeleton-related curvature-sensing proteins can provide the roadmap for designing novel antibiotics capable of inhibiting critical membrane remodeling or cytokinesis processes. For instance, β-lactams such as cephalexin and piperacillin specifically inhibit the transpeptidase activity of penicillin-binding protein (PBP3, also known as FtsI), an essential protein for FtsZ-ring constriction (21).

Another example of a curvature-sensing protein in bacteria is polar chemoreceptor clusters. In *B. subtilis*, the chemoreceptor TlpA has been reported to localize to the cell poles and is recruited to negative-curvature membranes (22). The *Escherichia coli* serine chemoreceptor Tsr is also sensitive to membrane curvature; it forms a trimer of dimers, and its intrinsic shape is considered to have a higher affinity for curved membranes (23).

Although investigations on prokaryotic proteins that sense membrane curvature in organelle-like bodies or plasma membranes have gradually increased, most of the underlying molecular mechanisms remain unclear. *In vitro* assays are essential to understand these molecular mechanisms and quantitatively evaluate curvature sensitivity by excluding the effects of other proteins and molecules. Various *in vitro* assays have been developed recently for examining eukaryotic curvature-sensing proteins; however, these have been applied to only a few prokaryotic proteins. In addition, *in vitro* assays require sophisticated and laborious experimental procedures, which may be one reason for the relatively small number of curvature-sensing proteins reported from prokaryotes, and many such proteins are likely to remain undiscovered under this scenario. Consequently, a comprehensive exploration method for curvature-sensing proteins using proteomic analysis has been developed, which is applicable to prokaryotes (20, 24). Such novel exploration and evaluation techniques may expand our understanding of curvature-sensing proteins in prokaryotes.

Previously, Boekema et al. provided a detailed review of complex membrane structures and the proteins involved in their formation in prokaryotes, and Strahl et al. reviewed curvature-sensing proteins involved in membrane structure regulation (14, 25). However, these reviews primarily focused on the function of proteins in cells, with a limited description of analytical methods, particularly *in vitro* assays, for detecting the curvature-sensing ability of proteins. In the present review, we summarize the recent technological developments in *in vitro* and live cell assays for identifying curvature-sensing proteins, mainly in eukaryotes, as well as existing exploratory methods. We then review the known curvature-sensing proteins in bacterial cells, particularly from the perspective of analytical methods (Table 1), and discuss the challenges and prospects for future research on curvature-sensing proteins in prokaryotes.

**TABLE 1:** An overview of localizations and assays in proposed membrane curvature-sensing proteins in bacterial cells

Proteins	Bacterial species for curvature sensing study	Protein localizations	Membrane curvatures^*a*^	*In vitro* assays^*b*^	Live cell assays^*b*^
SpoVM	*B. subtilis*	Forespore	+	SLiC (26), SLB (27),	MO (26)
DivIVA	*E. coli, B. subtilis*	Cell division site	−		MO (18), CWD (28)
MreB	*E. coli*		−		MO (29), CWD (28)
TlpA (chemoreceptor)	*B. subtilis*	Cell pole	−		MO [21]
Tsr (chemoreceptor)	*E. coli*	Cell pole	−		MO (23), CWD (23)
MamY	*Magnetospirillum gryphiswaldense*	Magnetosome	+		MO (30)
McaA	*Magnetospirillum magneticum*	Magnetosome	+		MO (31)

^*a*^ +, positive (convex) curvature; −, negative (concave) curvature.

^*b*^ SLiC, single liposome curvature assay; SLB, supported lipid bilayer assay; MO, microscopic observation; CWD, cell wall deformation assay.

## EVALUATION AND EXPLORATION OF MEMBRANE CURVATURE-SENSING PROTEINS

### *In vitro* evaluation of curvature-sensing property

*In vitro* experimental approaches are crucial for elucidating how proteins preferentially interact with membranes of a specific curvature. To exclude the influence of other proteins that may recruit the target protein to curved membranes via different mechanisms, the binding assay needs to use purified proteins and curvature-controlled artificial membranes (e.g., supported lipid bilayers or size-fractioned liposomes). The experimental method should be selected appropriately considering the curvature of interest (i.e., nanometer scale or micron scale). In addition, it should be noted that the geometrical difference between two-dimensional (spherical) curvature in the cell poles and one-dimensional (cylindrical) curvature in the sidewall may influence the curvature-sensing property of the protein (32). In general, the liposome-based approach can produce only two-dimensional curvature and tubular-based for only one-dimensional, while supported lipid bilayer can produce both geometries depending on the design of the substrates. Here, we specifically focus on methodologies for building membranes with radii of several tens or hundreds of nanometers and measuring the affinity between the curved membrane and target protein (i.e., the number of proteins bound to the membranes). We discuss a few different types of *in vitro* assay platforms utilizing positively and negatively curved membranes, while disregarding assays for curvature induction properties, such as protein-mediated deformation of unilamellar vesicles.

#### Liposome sedimentation/flotation assay

The amount of proteins bound to liposomes of different diameters has been compared using sedimentation or flotation assays (6, 33, 34). To prepare membranes with different intrinsic curvatures, liposomes were resuspended in a buffer and sequentially filtered through polycarbonate membranes with different pore sizes (e.g., 0.8, 0.4, 0.1, and 0.05 µm). In the reported sedimentation assays, proteins were incubated with liposomes by ultracentrifugation before sedimentation. After sedimentation, the content in the pellet (bound proteins) and supernatant (unbound proteins) was measured using sodium dodecyl sulfate-polyacrylamide gel electrophoresis (SDS-PAGE) and Coomassie Blue staining (6, 34). For the flotation assay, the suspension of proteins and liposomes was adjusted to a 30% (wt/vol) sucrose concentration and overlaid with 25% (wt/vol) sucrose solution and buffer before centrifugation. Proteins associated with liposomes were collected from the top fraction, and the relative amounts of proteins in the top, middle, and bottom fractions were quantified using SDS-PAGE (34). In both assays, the protein content bound to liposomes of different sizes was plotted against the liposome radius, represented by the membrane pore diameter or hydrodynamic radius determined by dynamic light scattering (34).

Although the sedimentation assay is a faster and more versatile method for various buffers and requires fewer lipids and proteins, it has drawbacks in terms of reliability. Proteins may undergo aggregation or oligomerization in the absence of liposomes, which can lead to sedimentation (35). Furthermore, the efficiency of liposome recovery by sedimentation reduces with a decrease in liposome size (34). The flotation assay is less sensitive to aggregation; however, several bacterial proteins possess the ability to deform cellular membranes (36–39). Thus, it is challenging to distinguish whether the protein has a higher affinity for a specific cell membrane morphology or whether the membrane is deformed due to protein localization. Additionally, liposome sizes greatly vary, with particularly large vesicles housing small vesicles, and the size distribution is mostly non-homogeneous (6). Therefore, the discussion on preferential membrane curvatures of proteins should carefully consider the influence of size distribution or utilize other platforms such as single liposome curvature (SLiC) assay and supported lipid bilayers (SLBs). Despite these experimental precautions, liposome-based assays are still powerful tools for examining protein–membrane interactions and are widely used because of their simplicity.

#### Single liposome curvature assay

**Fig 1:**
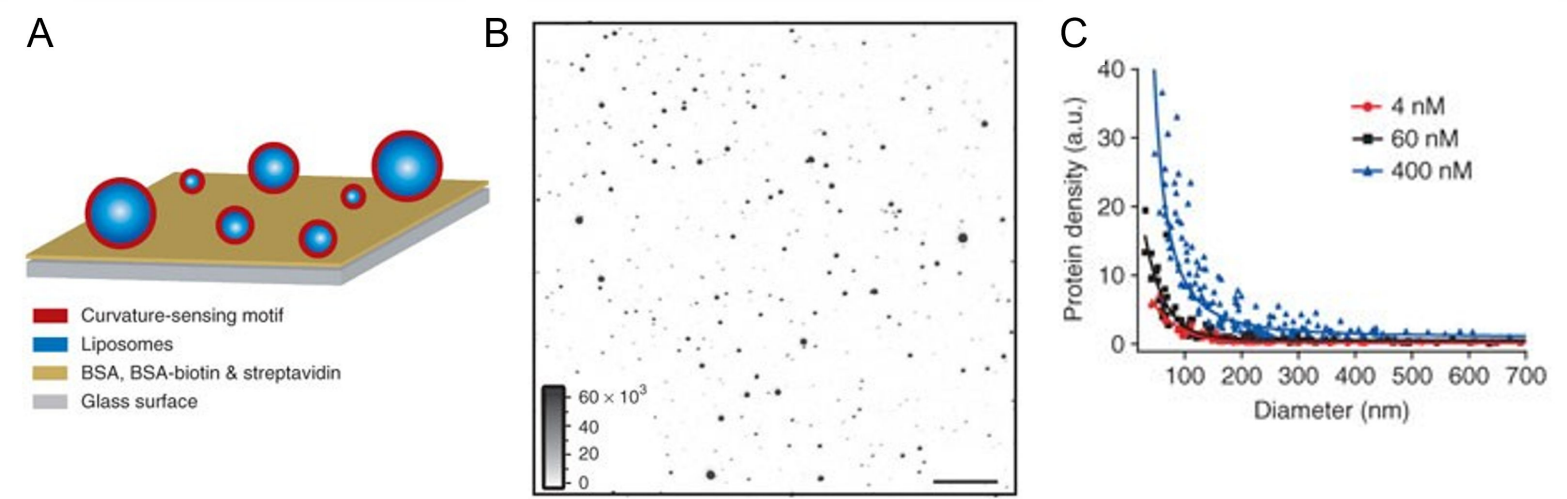
SLiC assay for evaluating curvature-sensing property. (A) Schematic illustration of SLiC assay with immobilized liposomes on substrate. (B) Representative confocal microscopy image of fluorescently labeled liposome. (C) The adsorption amount of endophilin amphipathic α-­helices domain against liposome diameter. Figure 1 is reproduced from reference 40.

To overcome the polydispersity of liposome sizes, biotinylated liposomes immobilized at low densities on a streptavidin-functionalized glass substrate have been utilized. Previous studies have monitored the interaction between proteins and isolated liposomes within a certain diameter range (50 nm–800 nm) in parallel using confocal fluorescence microscopy (40–42). This method, reported by Stamou’s group, was termed the single liposome curvature assay (40) (Fig. 1). In this study, liposomes were prepared by rehydration of lipid film containing a biotinylated lipid in D-sorbitol solution and were subjected to several freeze/thaw cycles in order to promote unilamellar vesicle formation before extrusion through a polycarbonate membrane with a pore size of 0.8 µm (41, 43). A glass slide was incubated with bovine serum albumin-biotin and streptavidin; thereafter, liposomes were applied and immobilized (44). The sizes of individual liposomes on the surface were accurately determined by integrating their fluorescence intensities (43). In addition, it was ensured that the liposomes were not deformed during the immobilization step by measuring the size of the contact area between the liposome and surface using fluorescence resonance energy transfer (45). Proteins labeled with a fluorescent dye (such as Alexa Fluor 488) or green fluorescent protein (GFP) were imaged with a second channel on a microscope, and the amount was evaluated (41, 46). The protein density of each liposome was plotted against the liposome diameter, and the SLiC assay was found to be more suitable for determining the real membrane curvature-sensing efficiency than bulk assays. This is because, when averaged out in ensemble sizes of liposome data, the apparent curvature-sensing efficiency decreases (42). The SLiC assay also provided insights into the nature of the binding of rat endophilin A1 1–247 (eNBAR) by comparing the curve of protein density and concentration on liposomes of different sizes; the data were fitted using the Langmuir isotherm. The membrane curvature preference of eNBAR is not due to its higher affinity toward small liposomes but due to its increased saturation density on highly curved membranes (42). The use of relatively larger vesicles (diameter 1 µm–30 µm) does not necessitate their immobilization on a glass substrate for confocal microscopic observation (26). This is attributed to the intrinsic sedimentation of vesicles, caused by the higher density of the internal solution in lower-density buffers.

The primary advantage of the SLiC assay is that each liposome has a unique and well-defined diameter; therefore, curvatures and hundreds of isolated liposomes with different curvatures can be analyzed simultaneously within a few seconds. This multipoint analysis enables the determination of the real curvature-sensing efficiency and quantitative discussion on the nature of curvature sensing. However, its drawback is that it needs careful consideration for use in investigating the mechanism of curvature preferences. While the SLiC assay or other liposome-based methods assume that the lipid compositions among vesicles are identical, it has been reported that there is significant heterogeneity in lipid compositions even when they are prepared using the same method (47). In addition, liposome-based assays can only examine interactions between proteins and biological membranes with a positive curvature.

#### Supported lipid bilayer assay

Another way to overcome the size polydispersity of liposome-based methods is to support the lipid bilayer with a solid substrate and then accurately define its shape. The simplest method for controlling membrane curvature is to use colloidal silica particles of varying diameters as substrates, which is called spherical SLB (SSLB). To evaluate the curvature-sensing protein SpoVM, localized on the surface of *B. subtilis* forespores, Ramamurthi et al. compared the amount of bound protein to small (2 µm) and large (8 µm) SSLBs (48). Briefly, dried *E. coli* polar lipid extract was rehydrated and sonicated to form liposomes. Afterward, a silica bead suspension and calcium chloride were added to the liposome solution, followed by incubation at 42°C to coat the bead surface with the lipid membrane. By comparing the fluorescence intensity per square micron of SpoVM-GFP on each SSLB using fluorescence microscopy, the protein was found to bind preferentially to a more convex membrane closer to the curvature of the forespore surface. It is also advantageous to use flow cytometry to measure protein quantity on SSLBs, which could increase throughput and minimize photobleaching concerns (49). Alternatively, and more conveniently, it is possible to incubate purified proteins with several differently sized SSLBs with the same total surface area and compare the amount of bound proteins for each SSLB using SDS-PAGE, which is similar to the liposome sedimentation assay (24).

However, because SSLBs use spherical substrates, they are only applicable for evaluating positive curvature and cannot be used to study negative curvature-sensing proteins such as DivIVA. To overcome this technical challenge, Hsieh et al. used wavy-shaped glass substrates instead of beads (50). By combining soft lithography and wet etching to form grooves with 110 nm depth and 1 µm pitch on a glass substrate, small unilamellar vesicles were fused on the glass substrate, and a lipid bilayer with positive and negative curvature was formed. The localization of fluorescently labeled ENTH and N-BAR domains on the membrane was observed by confocal fluorescence microscopy to evaluate the curvature-sensing property. In another study, the membrane curvature-sensing property of ESCRT-III protein was evaluated using a glass substrate with 100 nm deep invaginations with negative curvature (51). This negatively curved SLB technology can also be applied to study lipid localization at cell poles (52).

One drawback of these techniques is the lack of cytosol in SLBs. The thickness of the hydration layer between the substrate and lipid bilayer is a few nanometers, which may not sufficiently mimic living cells (53). Therefore, it is difficult to apply this method to study integral membrane proteins such as chemoreceptors, some of which are known for their curvature-sensing properties in prokaryotes (22).

#### Lipid nanotube assay

**Fig 2:**
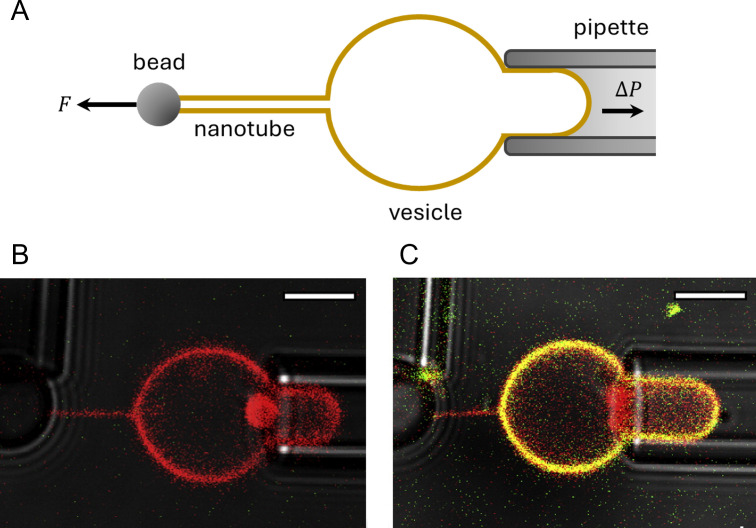
Lipid nanotube assay for observing the partitioning into curved membranes. (A) Illustration of experimental setup of nanotube extracted from a GUV. The cross-section of nanotube is highly curved compared with that of the vesicle. (B, C) Membrane labeled with Texas Red dye images taken before (B) and 18 min after (C) the addition of Alexa Fluor 488-labeled cholera toxin subunit B protein (scale bar: 5 µm). Figure 2 is reproduced from reference 54.

In the lipid nanotube assay, membrane nanotubes are extruded from micropipette-aspirated giant unilamellar vesicles (GUVs) using a bead folded by another micropipette or optical tweezers (54, 55) (Fig. 2). It can produce curved membranes ranging from several to hundreds of nanometers by controlling the pulling tension. Fluorescently tagged proteins and dye-labeled lipids are observed by confocal microscopy, and the enrichment of curvature-sensing proteins has been evaluated by comparing the protein- and lipid-derived fluorescence intensities in the vesicles and tubes (56, 57). Long and thin lipid nanotubes are also pulled from GUVs using kinesin motors (58, 59).

A clear advantage of this technique is its ability to precisely control membrane curvature. In addition, it can evaluate protein sensitivity to not only positive curvature but also negative curvature by adding proteins inside the vesicles (57). Furthermore, the curvature sensitivity of the transmembrane proteins tetraspanin 4 and CD9 can be studied using giant plasma membrane vesicles obtained from cells instead of artificial lipid membranes (56). However, membrane tension should be carefully considered, as it affects the alignment of curvature-sensing proteins (60).

### Live cell assays

Although *in vitro* assay platforms are becoming fundamental tools for studying curvature-sensing proteins, these artificial membranes cannot fully mimic the complexity of the phospholipid composition, glycans, and other membrane proteins present in living cell membranes. Therefore, the existing *in vitro* assays must be validated in living cells. Fluorescently tagged proteins are the most commonly used tool for visualizing protein localization. To eliminate the influence of factors independent of membrane curvature (e.g., the localization of some proteins and lipids), several techniques have been developed to deform the cell membrane into the desired shape. Here, we review some recent studies on living cell assays for curvature-sensing proteins, including the manipulation of membrane morphology.

#### Protein localization analysis by fluorescence microscopy

The localization of most curvature-sensing proteins in prokaryotes was first visualized by their fusion with GFP (18, 19, 22). Although GFP-based visualization has a strong advantage for clearly observing the intracellular localization of proteins, it should be noted that localization may not be caused by membrane geometric factors. To isolate the impact of membrane morphology on protein localization from other variables, researchers often conduct studies under conditions that induce morphological changes in the cell membrane. If proteins are capable of recognizing membrane curvature, their localization is expected to disappear when the curved membrane becomes uniform. Some researchers removed the cell wall of *B. subtilis* with lysozyme to obtain the protoplast and confirmed that DivIVA is uniformly distributed in the protoplast (19). For another curvature-sensing protein in *B. subtilis*, SpoVM, localized on the outer surface of the forespore, the localization of the GFP-tagged protein in the triple mutant strain with a straight polar septum was compared with that in wild-type cells (26). While these techniques lend support to the curvature hypothesis, some factors other than the shape of the cell membrane may be unintentionally altered. The evidence for the control of the cell membrane shape by external forces is more compelling.

#### Cell wall deformation in agarose microchamber

**Fig 3:**
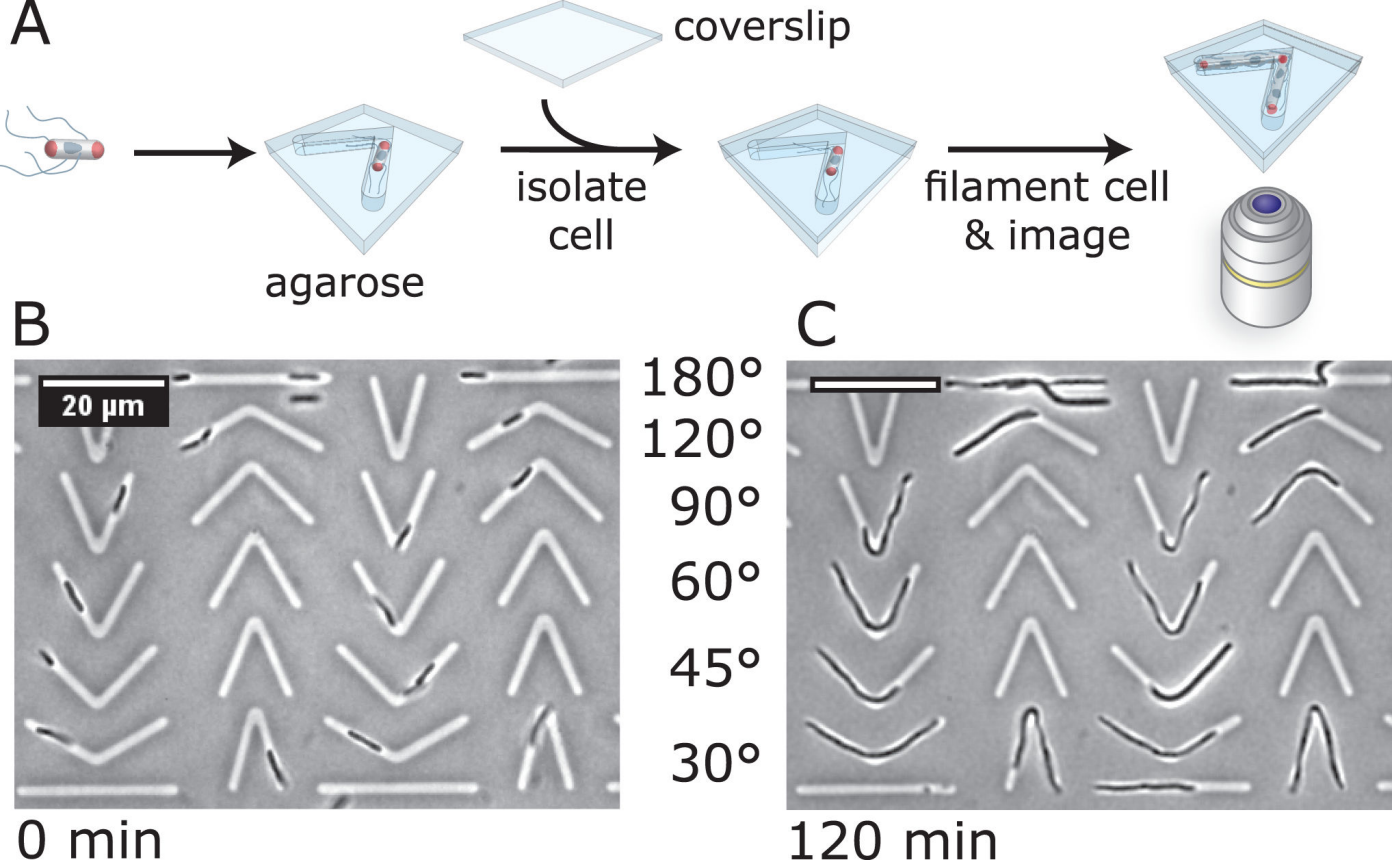
Microchannel-based methodology for controlling membrane curvature in bacterial live cell assay. (A) Schematic illustration for engineering the cell wall curvature of filamentous *E. coli* in “V”-shaped agarose microchannels. (B, C) Representative images of growing cells at 0 and 120 min. Figure 3 is reproduced from reference 28.

One method to deform microbes to create the membrane curvature of interest is to grow filamentous cells in an agarose microchamber with a defined shape (Fig. 3). A microchamber is typically fabricated by replica molding against a poly(dimethylsiloxane) stamp (61). The cell suspension is added to an agarose pad, and the cells are spontaneously guided into the microstructures by capillary pressure. Next, filamentation of the bacterial strain is induced by the addition of cephalexin, which inhibits PBP3 of *E. coli*, or by genetic regulation with the *mciZ* gene, which encodes a 40-amino acid peptide that inhibits the assembly of the FtsZ ring in *B. subtilis* (28). Fluorescently labeled actin-like cytoskeleton protein MreB foci prefer to localize in positively curved membrane compared with negatively curved one in filamentous *E. coli* cultured in a V-shape microchamber. The preference of chemoreceptor clusters for negatively curved membranes has also been confirmed using filamentous *E. coli* in agar microchambers (23).

This method can artificially deform cells into arbitrary shapes to observe the effect of their morphology on protein localization while maintaining the complex cell membrane composition of the original cell. The largest value of mean curvature imposed using this technique, however, is approximately 1 µm^−1^, which does not match the cell pole or division septa of bacterial cells (2–10 µm^−1^). Therefore, researchers have utilized spheroplasts or protoplasts instead of living cells to create more convex membranes when studying the cell division protein DivIVA (28).

#### Nanostructure assay

**Fig 4:**
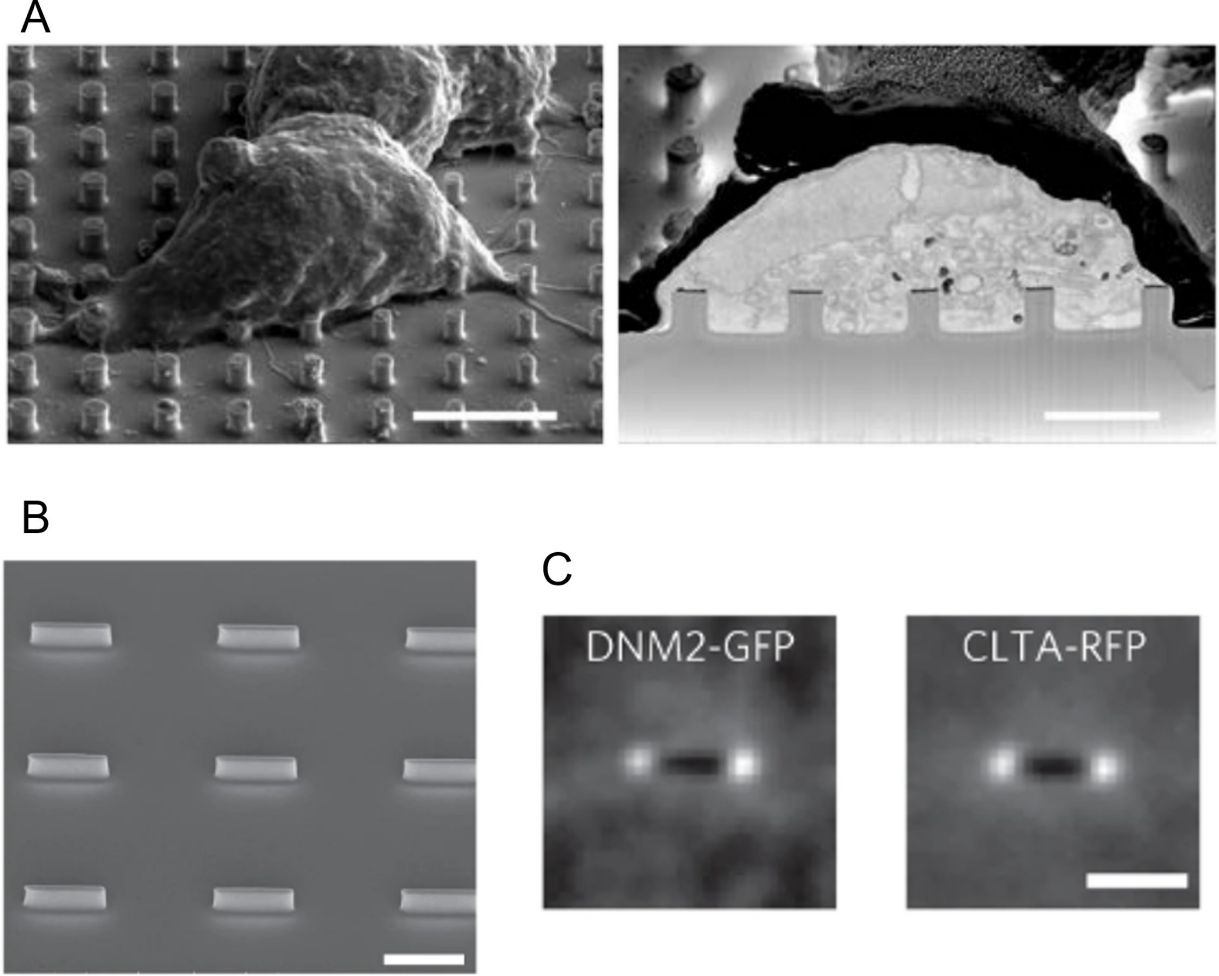
Nanostructure assay for localization of fluorescence proteins in a live cell. (A) Scanning electron microscopy (SEM) image of cell–nanopillar interface (left) and cross-section view after forced ion beam milling (scale bar: 10 µm). (B) SEM image of nanobar array (scale bar: 2 µm). (C) Averaged images of fluorescently tagged dynamin2 (DNM2) and clathrin light chain A (CLTA) localization on 167 nanobars (scale bar: 2 µm). Figure 4 is reproduced from reference 62.

The shape of the bottom cell membrane of adherent cells can be precisely manipulated by culturing them on a nanopillar substrate fabricated using electron-beam lithography and reactive ion etching (62, 63). In one study, several proteins including the N-BAR domain superfamily related to clathrin-mediated endocytosis were investigated in cells on a vertical nanostructure array, and it was confirmed that their localization was induced by external deformation (62) (Fig. 4). In addition to nanopillars, cone-shaped tin oxide nanostructures (nanocones) and other vertical nanoscale structures have also been developed and utilized in recent years (64, 65). These are powerful tools for creating well-defined membrane shapes and could provide convincing evidence that proteins preferentially accumulate in membranes with high curvature. However, while nanostructure-based manipulations require cells to adhere to the substrate, the deployment of this technique to study bacterial membranes remains challenging.

### Exploration technique

**Fig 5:**
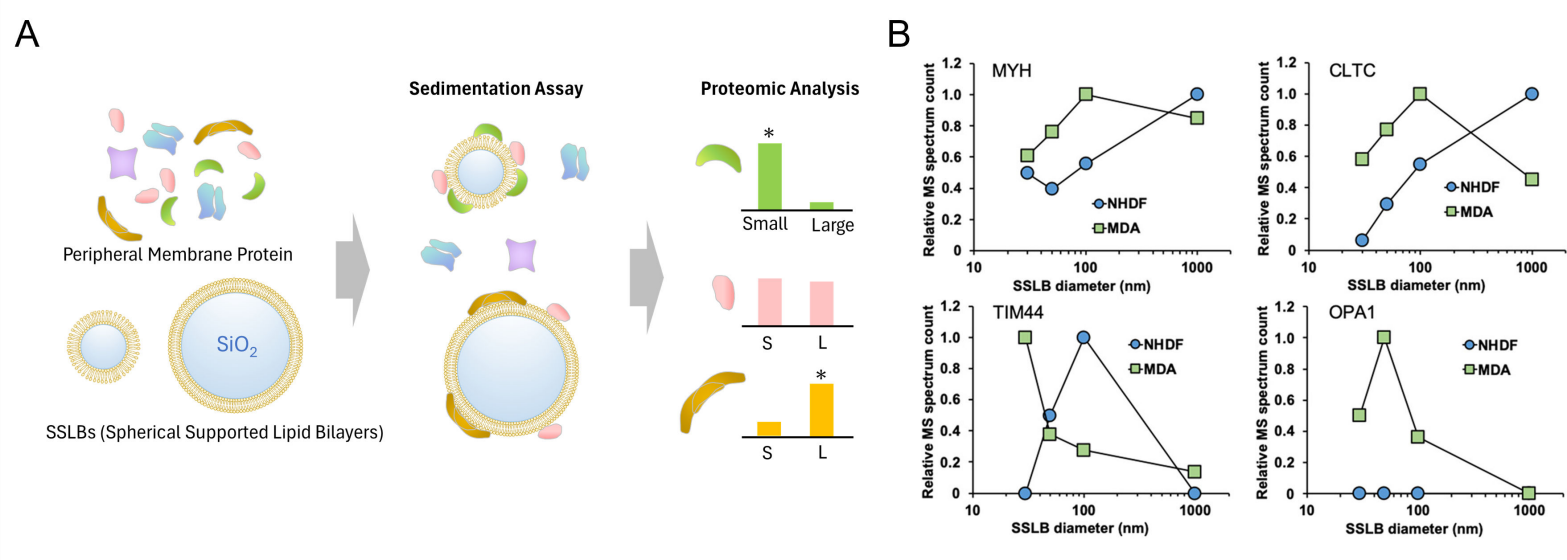
Exploration of curvature-sensing proteins by SSLBs and proteomic analysis. (A) Schematic illustration of the sedimentation assay and the statistical analysis for screening membrane curvature sensors. (B) The binding amount comparison of representative candidate proteins on a series of SSLBs with diameters ranging from 30 to 1 µm. Figure 5B is reproduced from reference 24.

Although various *in vitro* and live cell assays have been developed over the past few decades, as described above, evaluating curvature-sensing properties remains laborious. Only a few promising candidate proteins have been subjected to careful curvature-sensing testing, which requires the preparation of purified or fluorescently tagged proteins. Therefore, the identification of candidate proteins is crucial. In one study, researchers selected Snx33 as a candidate curvature-sensing protein in differentiated HL-60 cells, which have highly curved structures at the leading edge, by comparing the expression levels of BAR domain proteins between undifferentiated and differentiated cells using RNA-seq (66). However, elevated protein expression in differentiated cells with complex membrane morphologies does not always indicate that a protein possesses curvature-sensing properties. In some proteins, such as BAR domains, their high-dimensional structure is considered important for curvature sensing, while many intrinsically disordered proteins can also detect membrane morphology with different mechanisms (46, 67). Thus, considering the potential of curvature-sensing proteins from a structural perspective is challenging. A more straightforward way for screening curvature-sensing proteins was reported by combining the SSLB assay with proteomic analysis technology (24) (Fig. 5). In this method, the peripheral membrane protein fraction extracted from cells was incubated with SSLBs of four different sizes. Bound proteins were then identified and quantified by mass spectrometry-based proteomic analysis. Curvature-sensing proteins were selected by statistical comparison of the amount of bound proteins on small and large SSLBs. This method has also been applied to the prokaryote *E. coli* to demonstrate the curvature-sensing properties of FtsZ and SecA (20). Despite its appeal for potentially identifying unknown curvature-sensing proteins, this technique has several limitations. Because the sample applied to the binding assay is a mixture of numerous proteins, it is not feasible to determine whether the candidate protein is capable of independently sensing membrane curvature or is merely recruited by other proteins. Therefore, further confirmation using *in vitro* assays with purified proteins is required. Moreover, there is currently no established methodology for the screening of transmembrane curvature-sensing proteins.

## MEMBRANE CURVATURE-SENSING PROTEINS IN BACTERIA

### SpoVM

SpoVM is a small (26 residue-long) protein produced in the mother cell chamber during sporulation of *B. subtilis*. This amphipathic α-helix protein fused with GFP localizes to the mother cell membrane that surrounds the forespore after asymmetric division (8). In 2009, Ramamurthi et al. reported that SpoVM can sense positively curved (convex) membranes through a binding assay of a purified protein with a heterogeneously sized population of unilamellar phospholipid vesicles (26). The preference for positive curvature was also confirmed by quantifying the adsorption of SpoVM onto SSLBs of different sizes (2 and 8 µm) using flow cytometry. This approach eliminates the effect of membrane tension caused by vesicle size (27, 49). The protein inserts the hydrophobic side of its amphipathic α-helix into slightly curved membranes, a feature thought to be critical for sensing membrane curvature. The localization is lost by the alanine substitution of proline at residue 9 (P9A), interrupting the α-helix structure (i.e., P9A has a longer α-helix chain than the wild type) (8, 48). However, studies have also proposed the capacity of this remarkably small protein to detect micron-scale membrane curvature. The off-rate, defined as the inverse of the residence time, of SpoVM-Cy3 on 2 or 8 µm SSLBs has been measured using single-molecule fluorescence microscopy. The wild-type SpoVM exhibited slower off-rates on more convex membranes, while no significant differences were observed for the P9A variant (9). The flexible N-terminus resulting from P9 has been suggested to be pivotal for SpoVM membrane curvature discrimination. This was demonstrated by constructing variants in which proline was reintroduced at positions 7–13 in the P9A variant (49). Furthermore, SpoVM directly recruits and tethers SpoIVA *in vivo*, which is the major coat morphogenetic ATPase. Polymerization of SpoIVA on the spore surface prevents SpoVM from detaching from the curved membrane (9). The adsorption of SpoIVA onto SpoVM was assessed *in vitro* using SpoVM-coated SSLBs, thereby eliminating the complexity of living organisms (68).

### DivIVA

In *B. subtilis*, the approximately 170-amino acid protein DivIVA functions as an anchor that defines the site of cell division. DivIVA forms doublet rings at the mature division septa or at the site of ongoing cell division to recruit the cell division inhibitor MinC. This results in spatial separation of MinC and FtsZ, which allows cell division to progress between the two rings (17). In 2009, Lenarici et al. were the first to report that the localization of DivIVA to the cell division site may be attributed to its higher affinity for binding to negatively curved (concave) membranes (18). While there was no established *in vitro* method for testing binding to negatively curved membranes at that time, researchers investigated the localization of DivIVA in aberrantly shaped cells, including *B. subtilis* mreB mutant cells and *E. coli* murein hydrolase mutant cells. In another study, spherical cells were generated through enzymatic removal of the cell wall using lysozymes (19). The resultant protoplast, which exhibited uniform membrane curvature, was employed for evaluating the localization of DivIVA in comparison to wild-type cells. In both *B. subtilis* and *E. coli*, DivIVA was observed to localize to sites where the membrane was most strongly curved, whereas no localization was observed on the uniformly curved membranes of protoplasts, indicating that DivIVA possesses curvature-sensing properties. The higher affinity of DivIVA for negatively curved membranes can be explained by a “molecular bridging” mechanism (18). DivIVA is known to form doggy bone-like oligomers consisting of six to eight molecules (69). Clusters of these oligomers have fewer interactions with flat membranes and are more prone to detachment and diffusion. Conversely, clusters that bind strongly to curved membranes are more stable, as the exposed surface area is reduced. This hypothesis is further supported by Monte Carlo simulations of 200 spheres representing the DivIVA cluster in rod-shaped or hemispherical volumes.

### MreB

MreB is a bacterial actin homolog (cytoskeletal protein) that has been observed to form filaments of varying lengths, from 250 nm to longer filaments, encircling the cell periphery by more than half a turn. These filaments facilitate the maintenance of the rod shape by moving underneath the cell wall (36, 70). MreB filaments possess a curved ultrastructure and interact with the membrane on the outer surface of its bend (71). To determine whether MreB localization occurs in a curvature-dependent manner, protoplasts expressing GFP-MreB were grown on agar pads and found to exhibit micropatterns of varying widths (2 µm–5 µm) (36). As the difference in the principal curvatures decreased, the alignment of MreB filaments became increasingly disordered, indicating that MreB filaments have a preference for alignment on the circumference of the rod rather than on a spherical surface. In a separate study, the filamentation of *E. coli* was induced by treating the cells with cephalexin, an inhibitor of the cell division protein FtsI. Time-lapse epifluorescence imaging showed that MreB was concentrated on the inner aspect of the curved wall in S-shaped, elongated *E. coli* (29). Additionally, MreB-RFP localization in filamentous *E. coli* with a V-shaped cell wall has been observed in agarose microchambers (28). These findings are in agreement with the localization of MreB on the side of rod-shaped cells, where the difference in principal curvature is the greatest. It is postulated that MreB filaments preferentially orient toward the greatest membrane curvature, promoting the propagation of rod shape in the active feedback process of cell straightening (29, 36).

### Chemoreceptors

Bacteria utilize a chemotaxis system to sense their surrounding environment. This system comprises a ternary complex of transmembrane chemoreceptors, an adaptor protein (CheW), and a histidine kinase (CheA). This sensory cluster is localized at the cell pole of many bacterial species (72, 73). The underlying mechanism of the polar targeting of these clusters has been investigated in the past few decades. Some studies have proposed that new clusters assemble at lateral sites and are stochastically localized at the sides and poles. Owing to multiple rounds of cell division, clusters are transported to the cell pole. Once clusters become polar, their positions may be constrained because of the energetically stabilizing effect of membrane curvature on large receptor complexes (74, 75). In contrast, other studies have suggested that the positioning of chemoreceptor clusters is influenced by membrane curvature (22, 23, 76). A study analyzed the localization of chemoreceptor clusters in filamentous *E. coli* grown in a curved agar microchamber and found that larger clusters are more likely to be enriched in highly curved regions (23). Curvature-dependent localization is hypothesized to be a consequence of the intrinsically curved shape of the chemoreceptor trimer of dimers (ToD). The tripod-like ToD shape exhibits energetic stability in convex membranes. Thus, when mutations that inhibit trimerization or rigidity of dimers are introduced, a loss of preference for curved membranes is observed (22). However, the contribution of membrane curvature to the mechanism of chemoreceptor localization at the cell pole is still under debate, and the involvement of other factors is possible (77, 78).

### MamY and McaA

Magnetotactic bacteria form magnetosomes, which contain ferrimagnetic nanoparticles and organize them into chains to detect the magnetic field lines of the Earth’s magnetic field. Magnetosome chains are positioned at the midcell with a connection to the actin-like MamK filament through MamJ protein (79, 80). MamY is also essential for localizing the magnetosome chain to the positively curved cytoplasmic membrane in *Magnetospirillum gryphiswaldense* (30). This protein exhibits weak homology to Centaurin BAR and has been shown to interact with membranes and tubulate liposomes (11, 81). In contrast, in *Magnetospirillum magneticum*, mamY deletion does not affect chain organization (11). Recently, it has been reported that McaA localizes to the positive inner curvature and serves as a landmark for magnetosome chains in *M. magneticum* (31). However, the mechanisms by which MamY and McaA detect positive curvature in their inner membranes remain unclear.

## CONCLUDING REMARKS

The mechanism by which bacterial cells regulate protein localization is a central topic in the field of bacterial biology. The existence of curvature-sensing proteins with a higher affinity for curved membranes has only recently been revealed (14, 26, 82). It is becoming increasingly apparent that these curvature-sensing proteins serve as anchors for the recruitment of other proteins to specific sites. However, understanding how these proteins “sense” membrane geometry remains a challenge. Although numerous mechanisms have been proposed, most are based on observations of phenomena and do not sufficiently engage with the quantitative discussion of the molecular mechanisms underlying these events. To ascertain the effect of membrane geometry on the intracellular localization of proteins, a series of *in vitro* assays have been designed employing artificial lipid bilayers and purified proteins. Additionally, live cell assays are a powerful tool for reproducing the complexity of living cells. Nevertheless, a convincing evaluation of the contribution of membrane curvature requires sophisticated experimental designs and techniques. Thus, the number of known curvature-sensing proteins is limited, and considering the broad spectrum of bacteria, it is likely that there are many unknown curvature sensors. A method for screening curvature-sensing proteins based on the quantitative comparison of protein adsorption on spherical artificial membranes has recently been developed. However, the development of technologies for identifying proteins that sense negatively curved membranes and transmembrane proteins is crucial. To overcome these challenges, negatively curved SLBs including lipid bilayer on wavy glass substrate or supported proteoliposomes may be useful. Informatics, combined with advanced experimental technologies, will play an increasingly important role. Recently, a novel design method for curvature-sensing peptides using evolutionary algorithms and molecular dynamics simulations has been reported (83). Considering the recent development in high-performance computing and machine learning technology, *in silico*-based approaches could be another streamlined method to discover and understand curvature-sensing proteins. Furthermore, a technique for examining complex protein networks is required to understand the mechanisms underlying these biological processes. Several proteins are thought to be involved in cell membrane remodeling. For example, DivIVA regulates positioning of the FtsZ ring by recruiting MinC, which inhibits FtsZ during cell division (17). Network analysis offers insights into the intricate mechanisms governing membrane remodeling, paving the way for further research. To examine the protein network, it may be beneficial to utilize an online database such as STRING and integrate informatics with the experimental approach (84). The emergence of these sophisticated and systematic technologies for the comprehensive exploration of curvature-sensing proteins will ultimately contribute to the expansion of the research field of bacterial biology.
